# On-demand orbital maneuver of multiple soft robots via hierarchical magnetomotility

**DOI:** 10.1038/s41467-019-12679-4

**Published:** 2019-10-18

**Authors:** Sukyoung Won, Sanha Kim, Jeong Eun Park, Jisoo Jeon, Jeong Jae Wie

**Affiliations:** 1 Department of Polymer Science and Engineering, Inha University, 100 Inha-ro, Michuhol-gu, Incheon, 22212 Republic of Korea; 2 Department of Mechanical Engineering, Korea Advanced Institute of Science and Technology, 291 Daehak-ro, Yuseong-gu, Daejeon, 34141 Republic of Korea; 3 World Class Smart Laboratory (WCSL), Inha University, Incheon, Republic of Korea

**Keywords:** Magnetic properties and materials, Nanoparticles, Colloids, Polymers, Nanoscale materials

## Abstract

Magnetic soft robots facilitate the battery-free remote control of soft robots. However, parallel control of multiple magnetic robots is challenging due to interference between robots and difficult maneuvers. Here we present the orbital maneuvering of manifold magnetic soft robots. Magneto-induced motion (magnetomotility) that includes the hierarchy of rotation and resultant revolution allows for the independent control of the robot’s velocity and orbital radius. The soft robot achieves a speed of 60 body length (BL) s^−1^, which is approximately 50, 000 times faster with 1/7 the weight of the current lightest legged soft robot. The hierarchical magnetomotility is suitable for versatile locomotion such as stairs and uphill climbing, underwater and above water swimming. Owing to their swimming functionality, a swarm of such soft robots is capable of transportation of cargo. On-demand orbital maneuvering of magnetic soft robots provides a new methodology for concurrent actuation of multiple robots exhibiting collective behaviors.

## Abstract (web summary)

Magnetic soft robots show advantages in wireless actuation, but normally demand complicated device preparations and three-dimensional magnetic manipulation. Won et al. show helical soft robots responsive to a magnetic field rotating along a single axis, whose velocity and orbits can be controlled independently.

## Introduction

The smooth-jointed motility of soft robots can be powered by various external stimuli such as thermal^1–4^, photonic^5,6^, pneumatic^7–10^, hydraulic^11^, hygroscopic^12–15^, combustive^16^, electrical^17^, and magnetic^18–24^ systems. Magnetic soft robots can be remotely manipulated owing to the high penetrability of the magnetic field and actuated at ambient temperatures without the requirement of heating or intermedia. Recent studies have demonstrated that a single magnetic soft robot can be steered through geometric mechanics^20,25,26^ and local body deformation by programming particle alignment^22^ and magnetic polarity^24^. Magnetic motility can be regulated via permanent magnet pathways^23^, rotational frequencies of Helmholtz coils^27^, and multi-axial direction of electromagnetic systems^19^. However, the soft robots are relatively slow in actuating across a large area owing to their small size. A majority of multi-robot operations have been limited to rigid body^28–31^ and colloidal particle^32,33^ systems. The untethered and battery-free motion control of soft robot groups^34^ remains a challenge due to the complicated coordination required for each independent movement.

Here, we present a novel approach for the manipulation of multiple soft robots regulated by a single axial magnetic rotation beneath the center of the substrate. Orbiting robots are designed for the proposed magnetic actuation. The designs of the robots are inspired by the celestial bodies in our solar system, which consists of multiple planets orbiting around the sun. Spinbot, which is an individual soft robot, hierarchically revolves around the center of the substrate by rotational movement. The hierarchical magneto-induced motion, i.e., magnetomotility, which comprises of rotational and resultant revolutional movements, generates an orbit of magnetic soft robots. The three distinct modes in the rotational motion, namely rotating, pivoting, and tumbling, are controlled by varying the speed of magnetic rotation. The orbital control via these three rotational modes enables the selection of desired orbits with independent velocities in the multi-body actuation. The orbital locomotion demonstrates the adaptability of the conformal movement that circumvents blocked pathways as well as magnetomotility in land and water environments for multiple, collectively soft robotic systems.

## Results

### Fabrication and characterization of orbiting magnetic soft robots

**Fig. 1:**
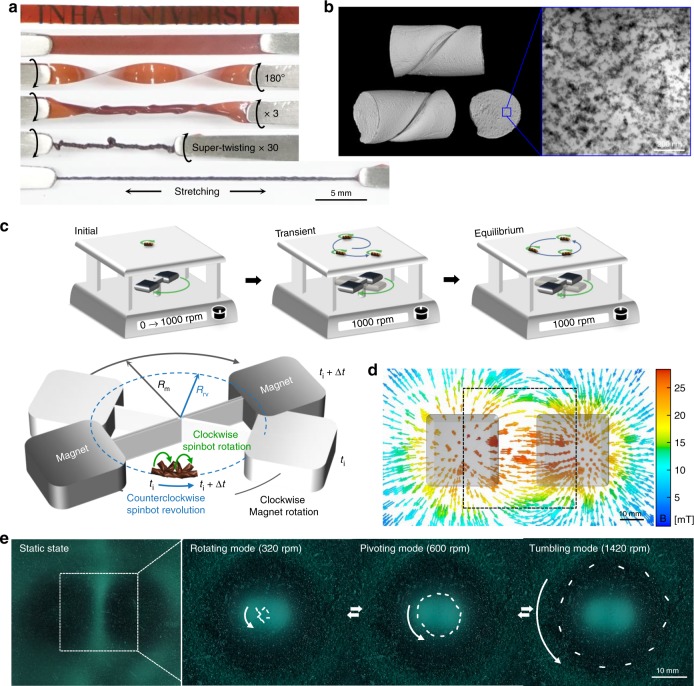
Facile construction of orbiting spinbots. **a** Digital image of 3D helical architecture generated from TPU-iron oxide nanocomposite film. **b** 3D micro-CT image of thermally treated helical nanocomposites (left) and TEM micrograph showing nanoscale dispersion of magnetic nanoparticles in TPU matrix (right). **c** Orbital maneuver of the spinbots via magnetic rotation. The spinbot rotates once clockwise for each magnetic rotation dependent on time Δ*t* from initial position at *t*_i_, revolving counterclockwise at equilibrium state. **d** Simulation of magnetic flux density involving two ferrite magnets with non-magnetic yoke of the magnetic stirrer. **e** Reversible control of orbital radius dependent on rotational speed

Our three-dimensional (3D) helical architecture is constructed for the magnetic soft robots by manually twisting stretchable (*ε* = 650%) thermoplastic polyurethane (TPU) nanocomposite films (Fig. 1a). Manual twisting is a facile, low-cost fabrication method in comparison to 3D printing^24^ or lithography^35,36^. The melt-pressed nanocomposite strips are supertwisted to avoid hollow structures and stretched. To attain dispersity of the magnetic nanoparticles in the polymer matrices (Fig. 1b, Supplementary Fig. 1), rapid precipitation method is employed. Transmission electron microscopy (TEM) micrographs confirm that homogeneous nanoscale dispersion of 10 wt% iron oxide nanoparticles (Fe_3_O_4_) is achieved, and the dispersion state of the polymer nanocomposite is quantified by local connected fractal dimension (*α* *=* 1.76) and lacunarity (*λ* *=* 0.09) in Supplementary Note 1. The helical architecture of the nanocomposite is fixed by thermal treatment at 175 °C for 20 min and cut into various aspect ratios (AR) of 1, 2, 3, 4, and 5, for soft robots with a diameter of 0.3 mm (Supplementary Note 2 and 3).

Rotating two ferrite permanent magnets on one axis, with *R*_m_ denoting the distance between the center of the magnet and the rotational axis, allows for the hierarchical magnetomotility of the 3D helical soft robots, i.e., a small radial rotation and large radial revolution (Fig. 1c). Once the two magnets rotate at the given speed and direction (e.g., clockwise), the soft robots containing nanoscale magnetic components follow the same rotational direction (clockwise). The rotational motion of soft robots corresponds to the number of calibrated rotations emanating from the magnetic source, as measured by a high-speed camera (Supplementary Fig. 5a). When rotating, the 3D helical geometry induces a minute mismatch between the geometrical center and the center of mass, and, therefore, it initiates a minor off-axis rotation. As the rotational speed increases, the rotating soft robots experience a lift force at one end and friction with the substrate at the other end, resulting in a revolution in the opposite direction (counterclockwise). On changing the clockwise rotating speed of the two permanent magnets from zero, the clockwise rotation of the soft robots is affected by the magnetic drag. The orbital radius of the helical soft robot, denoted by *R*_rv_, gradually increases until the orbital radius and velocity reach a constant equilibrium as a result of the balanced revolving centrifugal and magnetic centripetal forces. The spinbots occupy larger orbits at higher rotational speeds and the orbits are affected by the magnetic fields. This is confirmed by the overlapping trajectories in the magnetic viewing films as well as the simulated vector of magnetic fields shown in Fig. 1d and e. The magnetic field for the soft robot is horizontal to the surface at the center of the magnetic stirrer and inclines toward the magnets as the distance between the magnet and the soft robot reduces. Rotating 3D magnetic fields instigate multimodal modes of orbital rotation (Supplementary Note 4). The spinbots revolve between the two magnets with a magnetic flux density less than 0.03 T.

### Mechanism and simulation for regulation of orbital radius and velocity

**Fig. 2:**
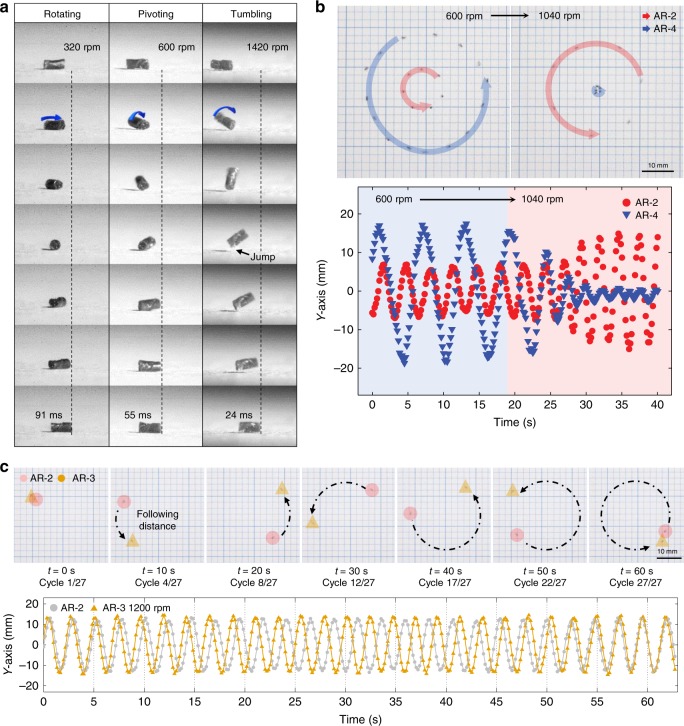
Regulation of orbital radius and velocity via rotational magnetomotility. **a** Evolution of three rotational modes of AR-2 amid half turn rotation upon magnetic speed manipulation. **b** Transferable orbital maneuver in a two-body system, including overlaid images (top) and *y*-axis displacement (bottom). **c** Time-lapsed trajectory showing that a faster spinbot catches up to a slightly slower spinbot via 27 orbital motions (top). The solid line in the graph refers to *y* = *R* sin(*θ*), when *R* is the average orbital radius of the spinbots and *θ* is the radian over time (bottom)

It is observed that orbital rotations (Fig. 2a) exhibit three distinct modes: rotating, where the two ends are mostly in contact with the substrate; pivoting, where displacement is observed at one end while the other end is fixed on the substrate, and the contact point with the substrate switches between 0 and 1; and tumbling, where pivoting occurs at a high inclined angle (>32°), which induces jumping when the contact points switch from one end to the other (Supplementary Movie 1).

The variation in the direction of the magnetic field at the static state is further investigated to provide lift angles for the soft robots on the substrate. At the center of the magnetic stirrer, the two edges of the spinbot are in contact with the substrate, and the lift angle increases up to 50° at 17.5 mm from the center of the stirrer (Supplementary Figs. 5b and 6). Thus, we conclude that the hierarchical magnetomotility, with respect to the orbital revolution, is affected by pivoting. In the equilibrium state, the orbital radius of the spinbots stabilizes at *R*_rv_, with a clockwise magnetic rotation speed of *ω*_m_. The pivoting spinbot of body length *l* travels a distance *s*_rv_ along the orbital perimeter during a single rotation, which can be expressed as

1 $$\frac{{2{\mathrm{\pi }}}}{{\omega _{\mathrm{m}}}} = \frac{{2{\mathrm{\pi }}}}{{\omega _{{\mathrm{rt}}}}} \approx \frac{{s_{{\mathrm{rv}}}}}{{v_{{\mathrm{rv}}}}} = \frac{{2l( {1 - \alpha _{{\mathrm{slip}}}} )}}{{R_{{\mathrm{rv}}}\omega _{{\mathrm{rv}}}}},$$

where *ω*_rt_ is the clockwise rotational speed of the robot (*ω*_rt_ *≅* *ω*_m_), *v*_rv_ and *ω*_rv_ are the velocity and counterclockwise angular speed of the orbital revolution, respectively. With respect to ideal pivoting, the contact point between the robot’s free end and the surface should be fixed by friction. Hence, *s*_rv_ is approximately twice the robot’s length, i.e., *s*_rv_ ≈ 2*l*. However, the robot slips on the substrates because the centrifugal force of the robot may exceed the friction. We define the probability of slippage *α*_slip_ according to its rotational speed as

2 $$\alpha _{{\mathrm{slip}}} = \frac{1}{2}\left[ {1 + {\mathrm{erf}}\left(\frac{{mr\omega _{{\mathrm{rt}}}^2}}{{F_{\mathrm{s}}}} - 1\right)} \right],$$

where erf() is the error function, *m* and *r* are the mass and half length of the rotating robot, respectively, and *F*_s_ is the static friction between the robot and surface. The probability of slippage is approximately zero at low rotational speeds $( {\omega _{{\mathrm{rt}}} \ll \sqrt {F_{\mathrm{s}}/mr} } )$ and increases as the rotational speed of the robot increases until friction cannot support pivoting $( {\alpha _{{\mathrm{slip}}} \cong 1\;{\mathrm{{when}}}\;\omega _{{\mathrm{rt}}} \gg \sqrt {F_{\mathrm{s}}/mr} })$. The force balance between the revolving centrifugal and magnetic centripetal forces, at equilibrium, can be described as

3 $$mR_{{\mathrm{rv}}}\omega _{{\mathrm{rv}}}^2 = mf_{\mathrm{m}},$$

where *f*_m_ is the magnetic attraction force per mass toward the center of revolution. Then, from Eqs. (1)–(3), based on the magnetic rotational speed and robot length, we can estimate the orbital radius and velocity as

4 $$R_{{\mathrm{rv}}} = \frac{{l^2\omega _{\mathrm{m}}^2}}{{4{\mathrm{\pi }}^2f_{\mathrm{m}}}}\left[ {1 - {\mathrm{erf}}\left(\frac{{ml\omega _{\mathrm{m}}^2}}{{2F_{\mathrm{s}}}} - 1\right)} \right]^2,$$

5 $$v_{{\mathrm{rv}}} = \frac{{l\omega _{\mathrm{m}}}}{{2{\mathrm{\pi }}}}\left[ {1 - {\mathrm{erf}}\left(\frac{{ml\omega _{\mathrm{m}}^2}}{{2F_{\mathrm{s}}}} - 1\right)} \right].$$

This relationship provides that the orbital maneuver of the spinbot is reversibly regulated (Supplementary Movie 2), and the lift angles induce three rotational actuations. Thus, we realize that multiple spinbots of varying body lengths can be steered simultaneously at distinct orbital radii and velocities by manipulating the speed of the magnetic rotation. AR-4 revolves at a radius of approximately 17 mm and 600 r.p.m., which is beyond the orbit of AR-2 which revolves at a radius of 5 mm, as shown in Fig. 2b (Supplementary Movie 3). This orbital configuration is reversed at 1040 r.p.m., where the orbit of AR-2 is greater than that of AR-4. Furthermore, it is possible to regulate each individual velocity of the spinbots traveling in subequal orbits (Fig. 2c). At 1200 r.p.m. of the magnetic source, the AR-3 spinbot, which rotates slightly faster, exceeds one full orbit of the AR-2 spinbot at the 27th cycle, which clocks in at 62.5 s (Supplementary Movie 4).

**Fig. 3:**
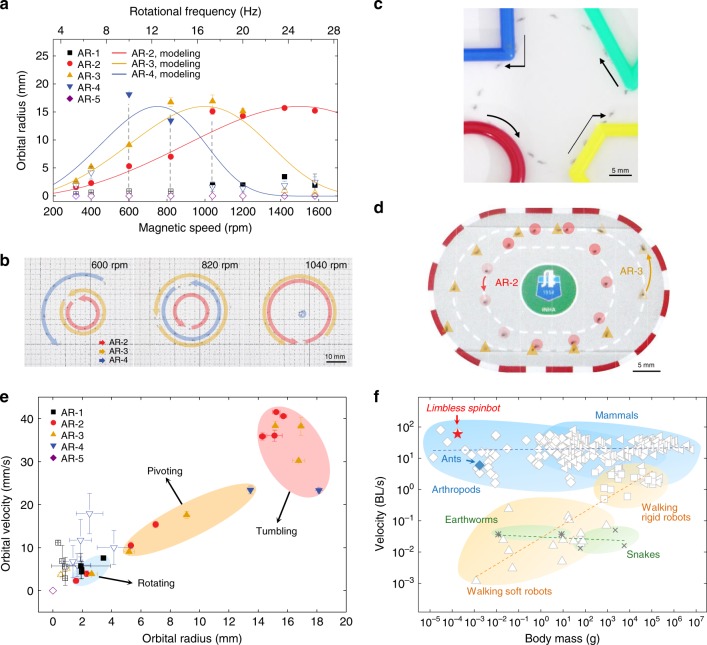
Maneuverability and adaptability of the agile spinbot. **a**, **b** Orbital radius control of the single spinbot (**a**) and three soft robots with different AR (**b**). Open symbols indicate non-uniformly revolving motions. **c**, **d** Conformal navigation of the single spinbot through various boundary conditions such as rectangular, diagonal, and circular edges (**c**) and two spinbots through two linear barriers (**d**) at 1040 r.p.m. **e** Phase diagram for three rotational modes. The fastest velocity of the spinbot is 42 mm s^−1^ in the tumbling mode. **f** Velocity normalized by the body length of the agile spinbot, walking soft robots, walking rigid robots, and living organisms. All error bars represent the standard deviation (*n* = 3)

The independent maneuverability of multiple soft robots with different AR is measured by the experimental data and compared with the model estimation. During the three-body manipulation, the orbital radius of each soft robot corresponds with that of the single body system (Fig. 3a, b, Supplementary Fig. 9, Supplementary Movie 5). The orbital maneuver of AR-2, 3, and 4 implies insignificant interactions between the robots because the orbiting is dominant at high-speed rotations.

### Multifunctional performance via orbital revolution

**Fig. 4:**
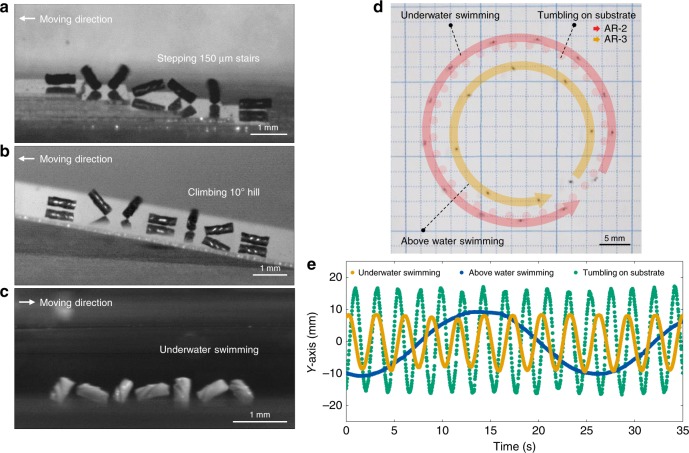
Multifunctionality of 3D helical spinbots. **a**–**c** Time-lapsed images of the revolving spinbots for 0.2 s. **a** Stepping AR-3 at 1040 r.p.m. **b** AR-3 climbing uphill at 1040 r.p.m. and overcoming slips by frictional force. **c** Underwater swimming of AR-2 at 1420 r.p.m. **d** Concurrent swimming motions of underwater AR-2 with an orbital period of 4 s and above water AR-3 with an orbital period of 19 s at 1200 r.p.m. The dot line is a guide of AR-2 tumbling on substrate. **e** Orbital maneuver of the amphibious spinbot in the case of AR-2 at 1580 r.p.m.

**Fig. 5:**
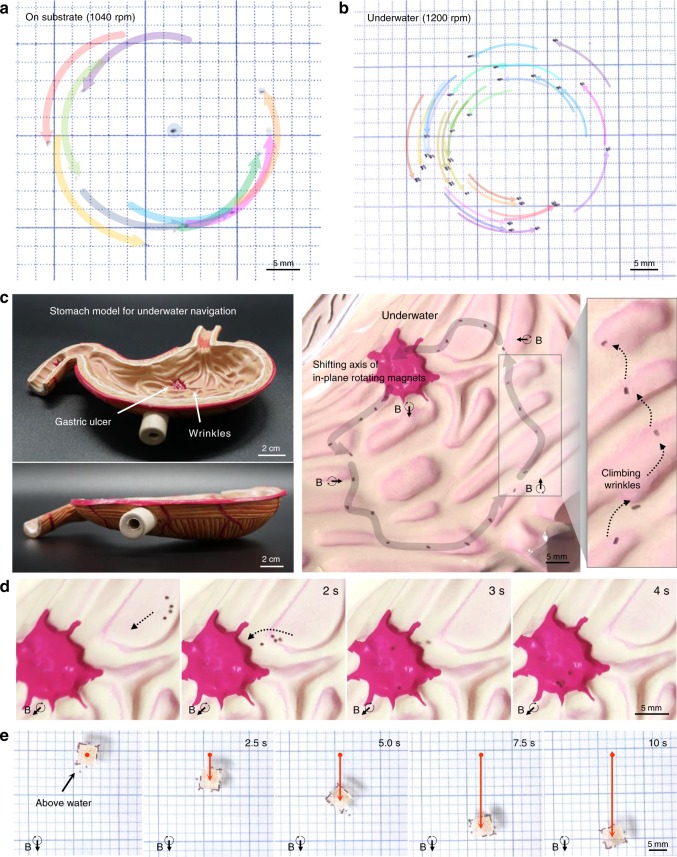
Multiple soft robots exhibiting collective behavior. **a** Ten spinbots actuated on the ground. **b** Underwater swimming of 37 spinbots actuated on the ground. **c** Underwater navigation of the single spinbot to a target gastric ulcer in the anatomical model of the stomach, for 20 s (1040 r.p.m.) and **d** multiple soft robots (1580 r.p.m.) through shifting axis of in-plane rotating magnets. **e** Transportation of cargo through collective motion of the spinbots above water as the axis of rotating magnets moves downward at a velocity of 2.3 cm s^−1^ (1040 r.p.m.)

When in-plane locomotive soft robots, i.e., caterpillar-like soft robots^23^, encounter obstacles in their path, three-dimensional regulation of external magnetic torque is necessary to avoid or climb over the obstacles. Hierarchical maneuver of the spinbots leads to conformal translational motility, and hence to the ability of circumventing obstacles along the various boundaries (Fig. 3c, d, Supplementary Movie 6) without the assistance of a programmed pathway or any artificial intelligence. Therefore, the orbital trajectories and velocities of these adaptable spinbots can be regulated concurrently during the navigation of a non-concentric pathway within linear boundary conditions, i.e., the running track. We further confirm that the three rotational modes govern the orbital radius and velocity of the spinbots through the construction of a phase diagram (Fig. 3e). The orbital radii and velocities are regulated by each rotational mode, excluding non-uniform orbital trajectory of the soft robots (open symbols). The velocity of the helical spinbot is compared with other robotic systems and living creatures normalized by their body length[13], as shown in Fig. 3f. Although the spinbots are legless, similar to earthworms^37^ and snakes^38^, they can move as fast as 60 BL s^−1^ with a body weight of 177 µg, which is comparable to the motility of an arthropod and 10 times faster than that of ants^39^. Compared to the walking soft robot that has the lowest weight^40^, the spinbot of AR-2 is approximately 50,000 times faster and 7 times lighter. The spinbots travel in an orbital trajectory that is up to 140 times larger than their own body length. The helical spinbots perform multifunctional acrobatic movements to navigate through various environments (Fig. 4, Supplementary Movie 7). The spinbot with a body length of 933 µm is capable of ascending 150 µm-high stairs. Although slips occur when the slope is greater than 10°, the spinbot can climb uphill up to a slope of 20°. The spinbots are capable of adapting to land and water conditions. While 2D films often float above water owing to surface tension and buoyancy, the void-free supertwisted soft robot can instantly immerse and navigate underwater through orbital rotation and revolution that can overcome surface tension. The spinbots are also capable of swimming in the reverse direction (Supplementary Fig. 13, see Supplementary Movie 9). Swimming robots have been propelled by the helical rotation of flagellated bacteria-like shapes^18,27,41^ and precession of achiral swimmers^42^. We propose multiple swimmable soft robots via hierarchical magnetomotility (Fig. 5, Supplementary Movie 8). In addition to the parallel magnetic control of three spinbots, the collective motion of 10 spinbots rotating on a substrate (Fig. 5a) and 37 spinbots swimming underwater (Fig. 5b) are achieved. The trajectory of the underwater swimming spinbots demonstrates reduced fluctuation owing to the presence of drag force. As shown in Fig. 5c, d, the spinbots are steered toward the gastric ulcer and successfully climb over the curved wrinkles of the stomach model. The orbital movement is reconfigured by altering the directionality of the magnetic field because the central axis of the orbits correlated with the axis of the rotational magnets. The rotating spinbots demonstrate translational motility when the applied rotating magnetic field is continuously relocated. In conjunction with this achievement in movement, the collective motion of the spinbots enables the transportation of cargo 100 times heavier (43 mg) than an individual spinbot (Fig. 5d). The cooperative nature in the operation of multiple spinbots is inspired by the collective quality of ant behaviors observed in nature^43^. To the best of our knowledge, this is the first reported case of the collective motion with parallel multimodal control of untethered polymeric multiple robots in contrast with use of rigid robots^28–31^. Although a single spinbot is unable to carry the object, the spinning force of the soft robots surrounding the cargo generates collective motion under the simplified external stimulus of planar magnetic rotation. This collective transportation motion is driven by capillary interactions^44^ between the cargo and the spinbots at the air–water interface, retaining rotational movement around the cargo.

## Discussion

The hierarchical magnetomotility of orbital rotation and resultant revolution is introduced by the facile magnetic manipulation of the rotating permanent magnets. The individual regulation of multiple soft robots is achieved by the use of trimodal rotations, which are switchable by simply varying the rotating speed of its magnetic source. During orbital revolution, pivoting and tumbling motion with agile velocity (up to 60 BL s^−1^) facilitates to circumvent barriers and climb over obstacles without regulation of magnitude and direction of magnetic force. We believe that the hierarchical actuation possesses great potential for the adaptive locomotion of soft robots in complex configuration space, such that, if combined with recent sophisticated multi-axial magnetic manipulation systems^19,45^, three-dimensional motion planning can be further programmed for miniaturized magnetic swimmers. The on-demand maneuvers of orbiting magnetic soft robots can serve as a triggered model to overcome the current challenges involved in untethered soft robotics such as low velocity, single modal actuation, and single object control.

## Methods

### Preparation of polymer nanocomposite robots

The rapid precipitation^46^ technique was employed to prepare polymer nanocomposites with nanoscale dispersion of iron oxide nanoparticles (Ferrotec Corporation, EMG1300 grade) in thermoplastic polyurethane (TPU) (KOLON INDUSTRIES, ELLAS K-185A grade, M_w_ = 257 kDa, PDI = 1.81) matrices at a 10 wt% nanoparticle concentration. TPU resins were fully dissolved in tetrahydrofuran (THF) for 7 days. Iron oxide nanoparticles were dispersed in THF by sonicating for 1 h. The TPU and iron oxide nanoparticle solutions were blended and sonicated for 5 min, followed by rapid precipitation into methanol, which is a poor solvent of TPU and iron oxide nanoparticles. The precipitated polymer nanocomposites were dried at 40 °C in a vacuum oven for 7 days to completely eliminate the residual THF.

The polymer nanocomposites were melt-pressed at 175 °C into 70-µm-thick films and cut into 20 mm × 2 mm strips. To acquire the helical shape of the magnetic soft robots, the two ends of the composite strip were simultaneously twisted with left-handed 180° rotations. The twisting was repeated 30 times to avoid hollow structures and followed by stretching the strips to 30 mm to exclude coiled structures. Subsequently, the strips were treated thermally at 175 °C for 20 min, followed by cooling at 25 °C to maintain an average helical angle of 30° in the 3D structures. The 3D helical soft robots were cut into AR of 1, 2, 3, 4, and 5, with a diameter of 0.3 mm.

### Orbital maneuver analysis

The orbital maneuver of the magnetic soft robots was analyzed by increasing the speed of the magnetic stirrer (Misung Scientific. Co. Ltd., HS180), beginning from the static states on the substrate. The magnetic stirrer contained two 30-mm-square permanent magnets (ferrite magnets, Y30) connected to a non-magnetic axis. The distance between the magnets was 18 mm, and the radius of the rotating magnet *R*_m_ was 24 mm. The coercivity *H*_c_ of Y30 was 200 kA m^−1^. The orbital radius was measured after the orbital revolution of magnetic soft robots was equilibrated on the stirrer. The magnetic flux density was simulated using Ansys aim.

## Supplementary information

Supplementary Information

Description of Additional Supplementary Files

Supplementary Movie 1

Supplementary Movie 2

Supplementary Movie 3

Supplementary Movie 4

Supplementary Movie 5

Supplementary Movie 6

Supplementary Movie 7

Supplementary Movie 8

Supplementary Movie 9

## Data Availability

The data that support the findings of this study are available from the corresponding author on reasonable request.
