# Supplementary material for: On-demand orbital maneuver of multiple soft robots via hierarchical magnetomotility
Source: Nat Commun. 2019 Oct 18;10:4751. doi: 10.1038/s41467-019-12679-4 (PMC6802085; doi:10.1038/s41467-019-12679-4)
Supplement: Supplementary file 1 — Supplementary Information [file 41467_2019_12679_MOESM1_ESM.pdf]

Supplementary Information:

On-Demand Orbital Maneuver of Multiple Soft Robots via Hierarchical Magnetomotility

Sukyoung Won[1], Sanha Kim[2], Jeong Eun Park[1], Jisoo Jeon[1], Jeong Jae Wie[1,3*]

[1]Department of Polymer Science and Engineering, Inha University, 100 Inha-ro, Michuhol-gu, Incheon 22212, Republic of Korea

[2] Department of Mechanical Engineering, Korea Advanced Institute of Science and Technology, 291 Daehak-ro, Yuseong-gu, Daejeon 34141, Republic of Korea

[3]World Class Smart Laboratory (WCSL), Inha University, Republic of Korea

*To whom correspondence should be addressed. E-mail:wie@inha.ac.kr

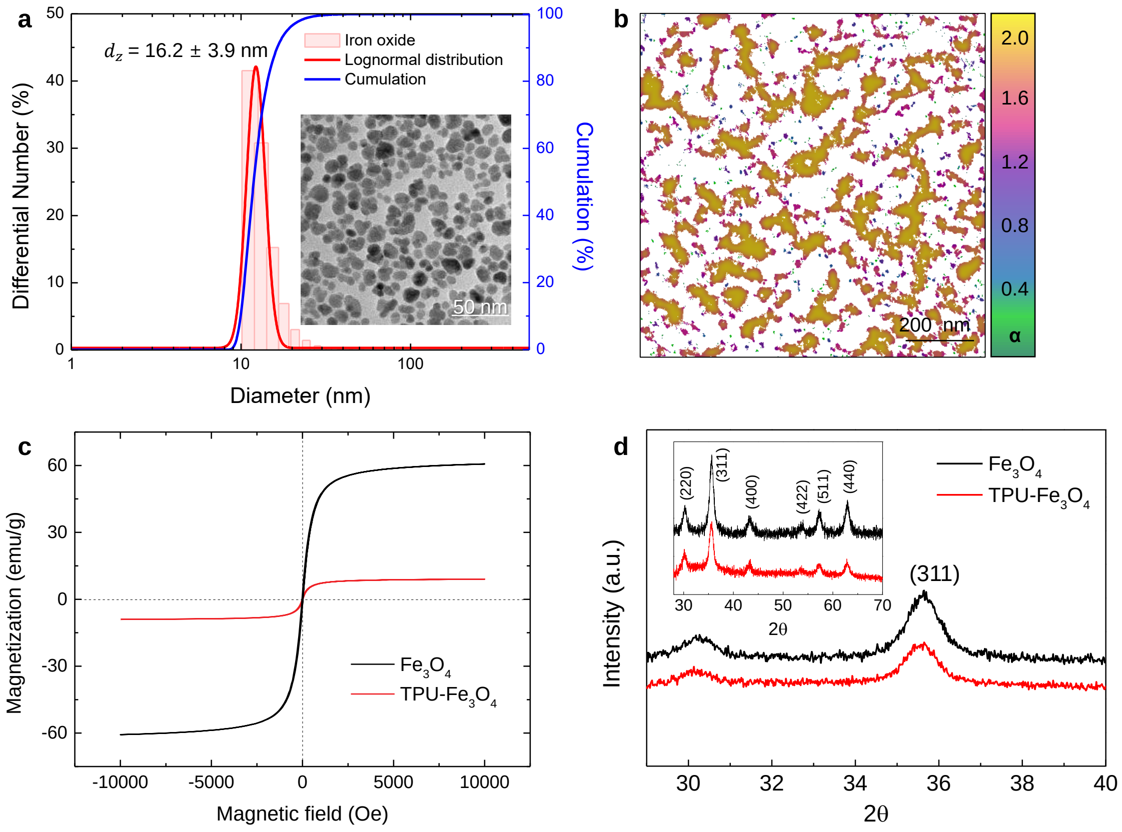

Supplementary Figure 1. Homogenously dispersed nanoparticles in the composite. (a) The z-averaged diameter of the iron oxide nanoparticles measured in THF by dynamic light scattering (DLS), inset image is the TEM micrograph. (b) Conversion of TEM micrograph of the 10 wt% nanocomposite in Fig. 1d to local connected fractal dimensional image. (c) Magnetization curves of Fe_3O_4 nanoparticles and 10 wt% Fe_3O_4 (TPU-Fe_3O_4) composite film are measured by vibrating sample magnetometer (VSM) at 300 K. (d) Peak intensity (311) of magnetite nanoparticles measured by X-ray diffraction (XRD).

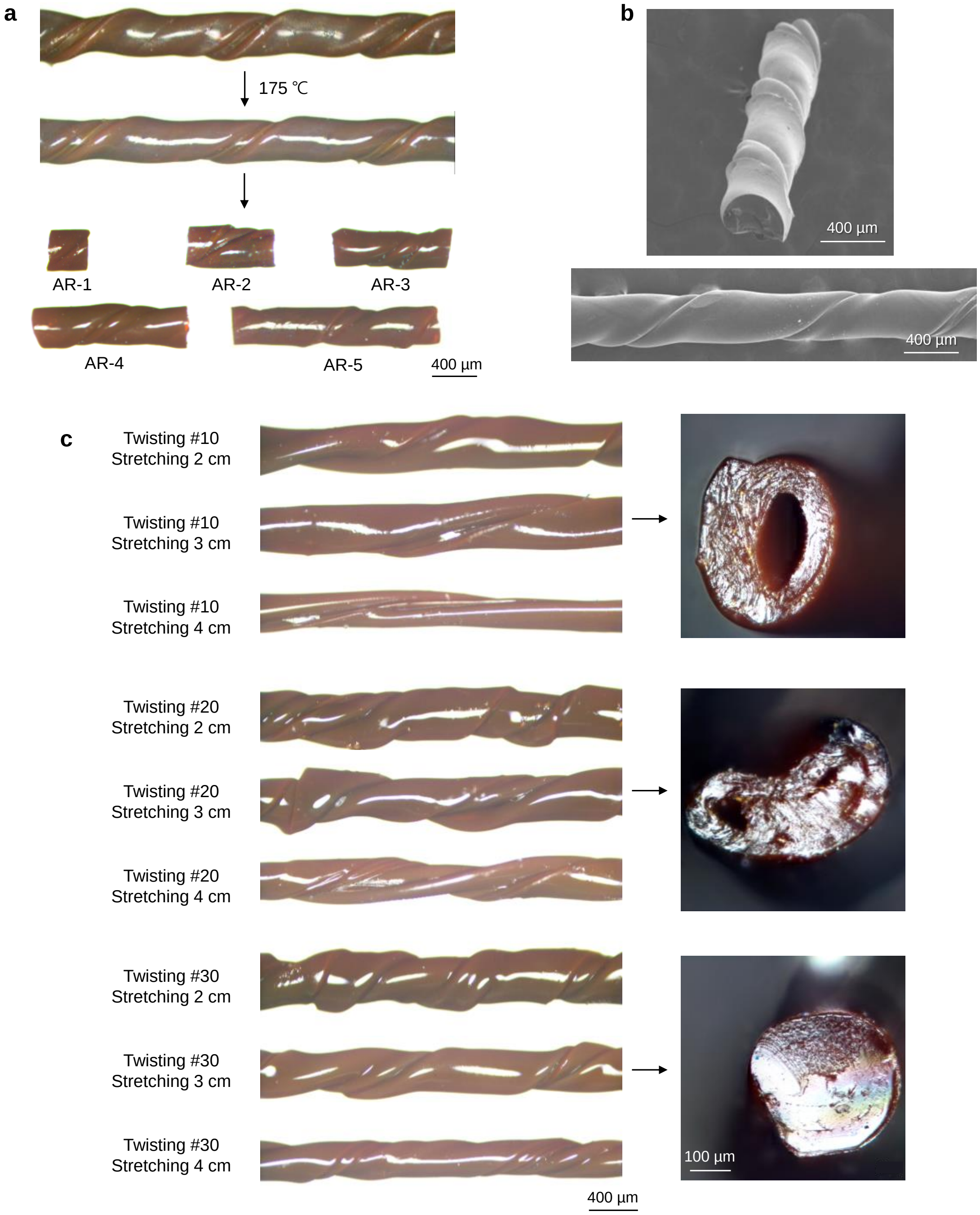

Supplementary Figure 2. Helical geometry of the nanocomposite. (a) Smoothing and fixation of 3D helical geometry by thermal treatment at 175 °C followed by cutting the spinbots with aspect ratio (AR) from 1 to 5. (b) SEM micrograph of 3D helical structure without voids. (c) The spinbots constructed from various twisting numbers (10, 20, and 30) and stretching lengths (2, 3, and 4 cm).

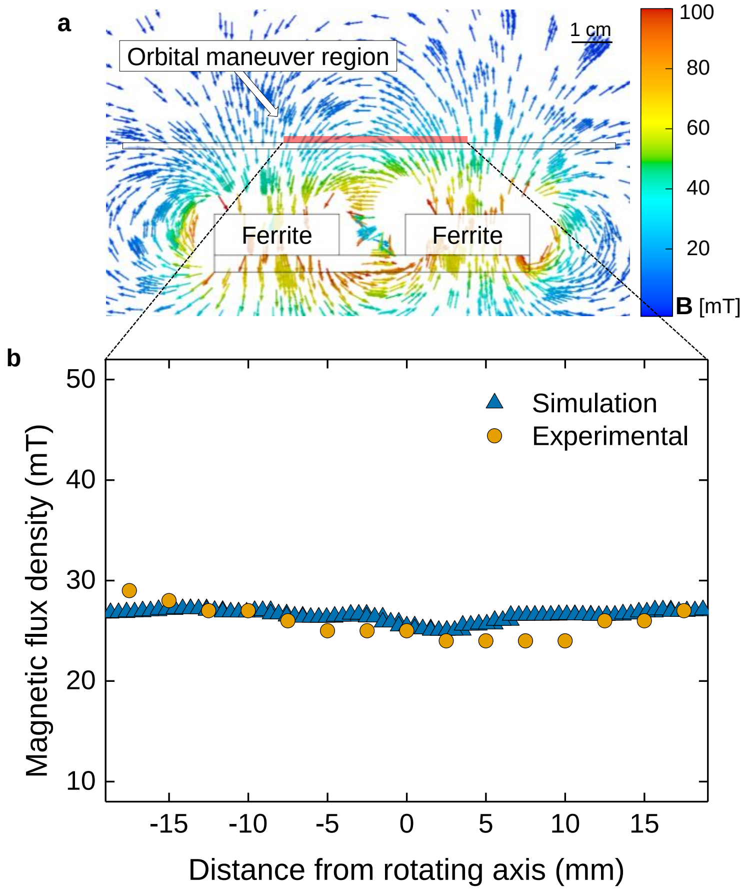

Supplementary Figure 3. Plot of magnetic flux density on the substrate. (a) Side view of simulated magnetic flux density near two magnets. The red region indicates area between the two magnets where the spinbot actuation occurs. (b) Comparison with experimental magnetic flux density by a gauss meter and simulation of (a). The spinbots revolve with orbital radius of up to 20 mm. The maximum value of the magnetic flux density is measured by placing the gauss meter in the identical vector direction that corresponds to the simulation.

The simulation of magnetic flux density shows the orbital maneuver region on the substrate of the magnetic stirrer programmed by Ansys aim. Rotating components of the stirrer involve two ferrite magnets (Y30) yoked by non-magnetic plate. Magnetic coercivity of ferrite is 200 kA m^{-1}. The dimension of each magnet is 30 mm x 30 mm x 20 mm. The center-to-center distance of the two magnets is 48 mm, and height is 18 mm from substrate to the magnets.

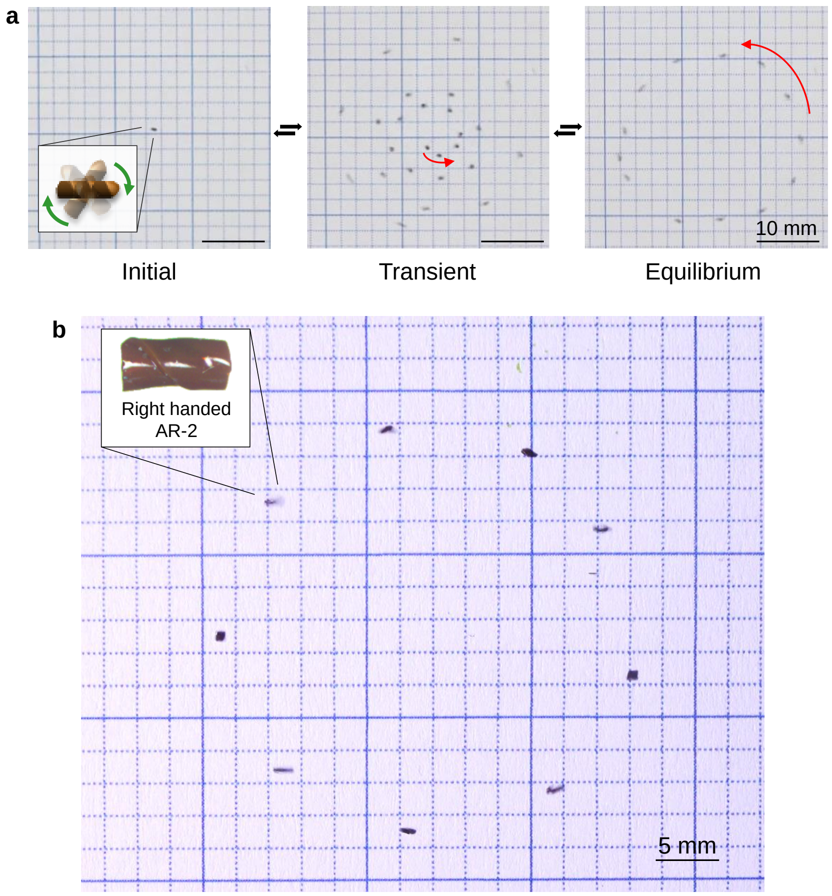

Supplementary Figure 4. Orbital maneuver. (a) Orbital equilibrium. Time-lapsed image of top-down orbital trajectory of the spinbots. (b) Orbiting right handed AR-2 at 1040 rpm. The 15 mm orbital radius is subequal with the left handed AR-2.

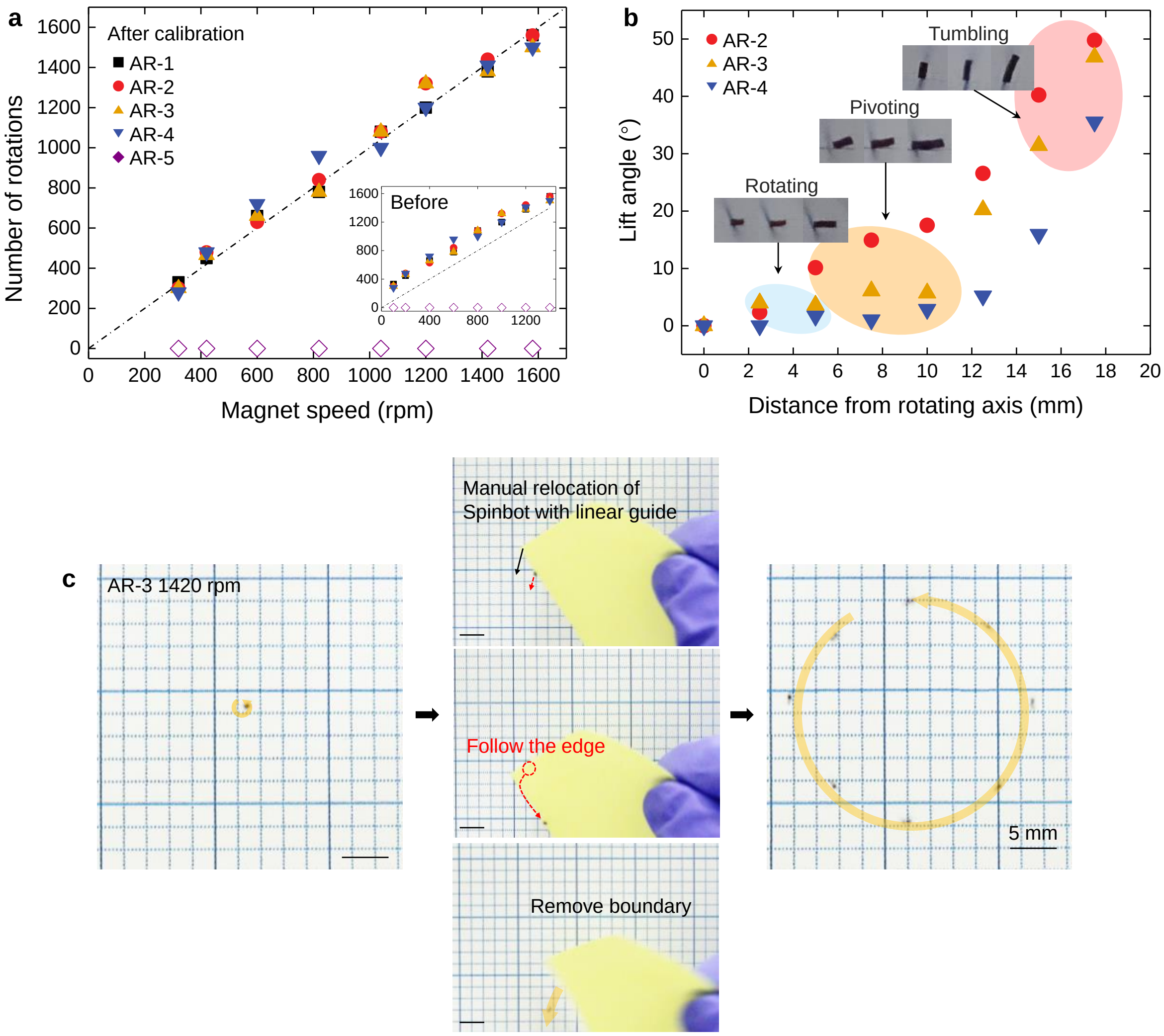

Supplementary Figure 5. Principles of orbital maneuver by magnetomotility. (a) Correspondence between number of rotations of the spinbots and calibrated magnet rpm. Insert graph is before calibration of magnet speed. AR-5 has no actuation. (b) Effects of magnetic field on static states of the spinbots. The spinbots standing with lift angles by the vector field on the stirrer in Fig. 1d resulted in three rotational actuations. The confined area of the three modes is divided in accordance with the measured orbital radius. (c) The conversion of rotational mode through manual relocation.

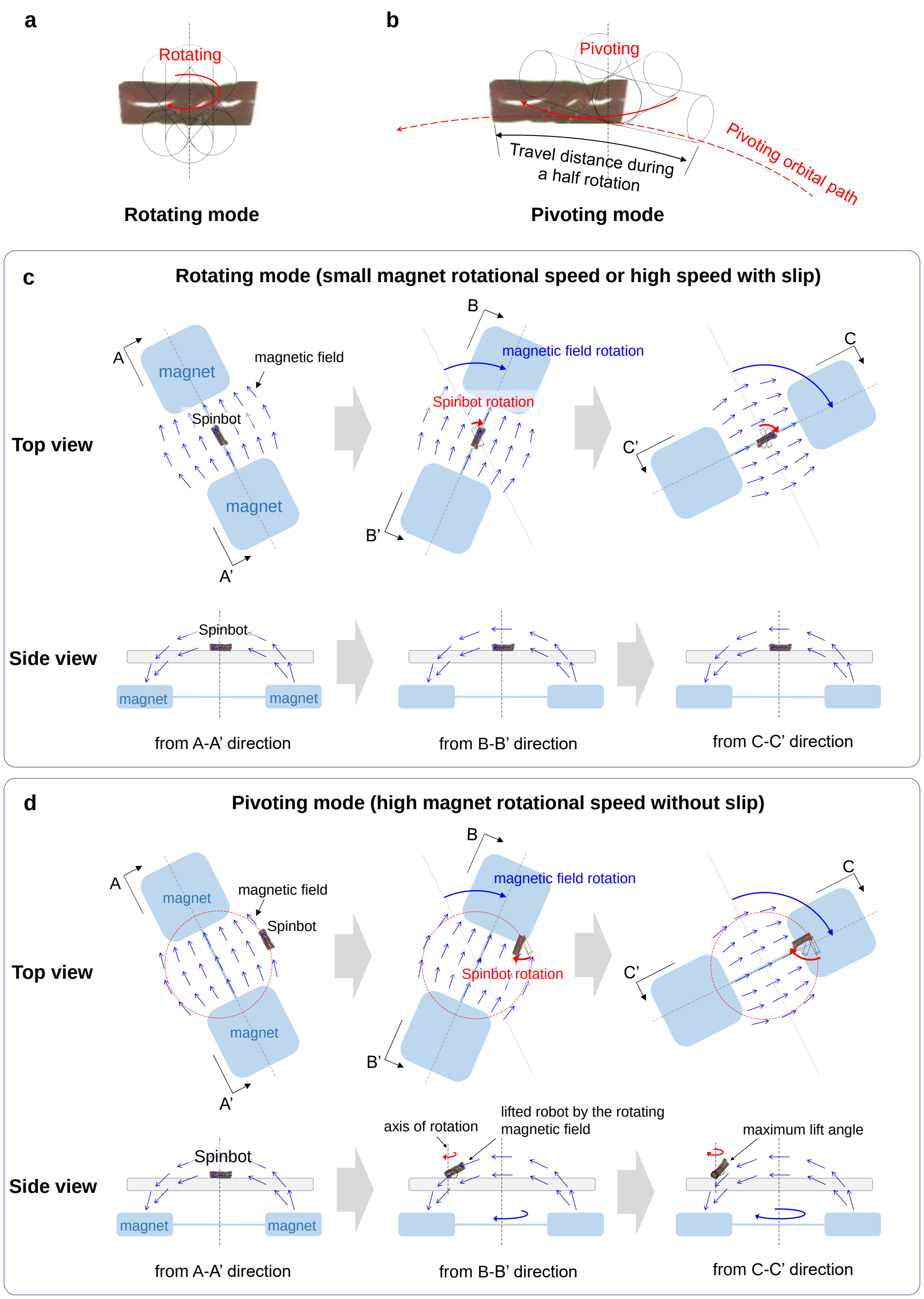

Supplementary Fig. 6. Orbital kinematics. Schematics for (a) rotating and (b) pivoting modes of the spinbots. Mechanism of (c) rotating and (d) pivoting at discrete moments of time under the 3D magnetic field in rotation. The side-views are from the view-points following the magnet during 90° of rotation; this shows the changes in both lateral and vertical directions of the magnetic field which generate a lift angle of the spinbot when the axis of rotation is off from the magnetic rotation axis.

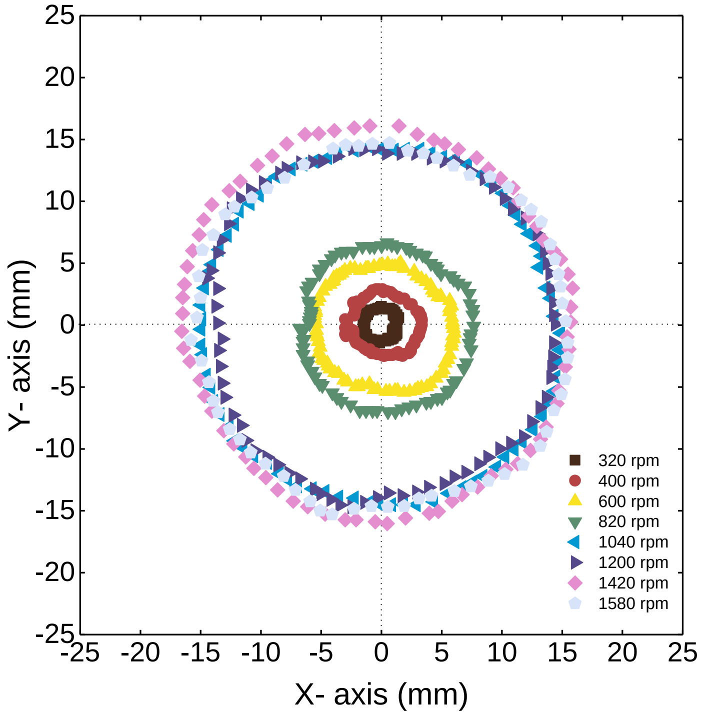

Supplementary Figure 7. Concentric trajectories in orbit. X and y-coordinate values of revolving AR-2 with various rotational speeds on the magnetic stirrer.

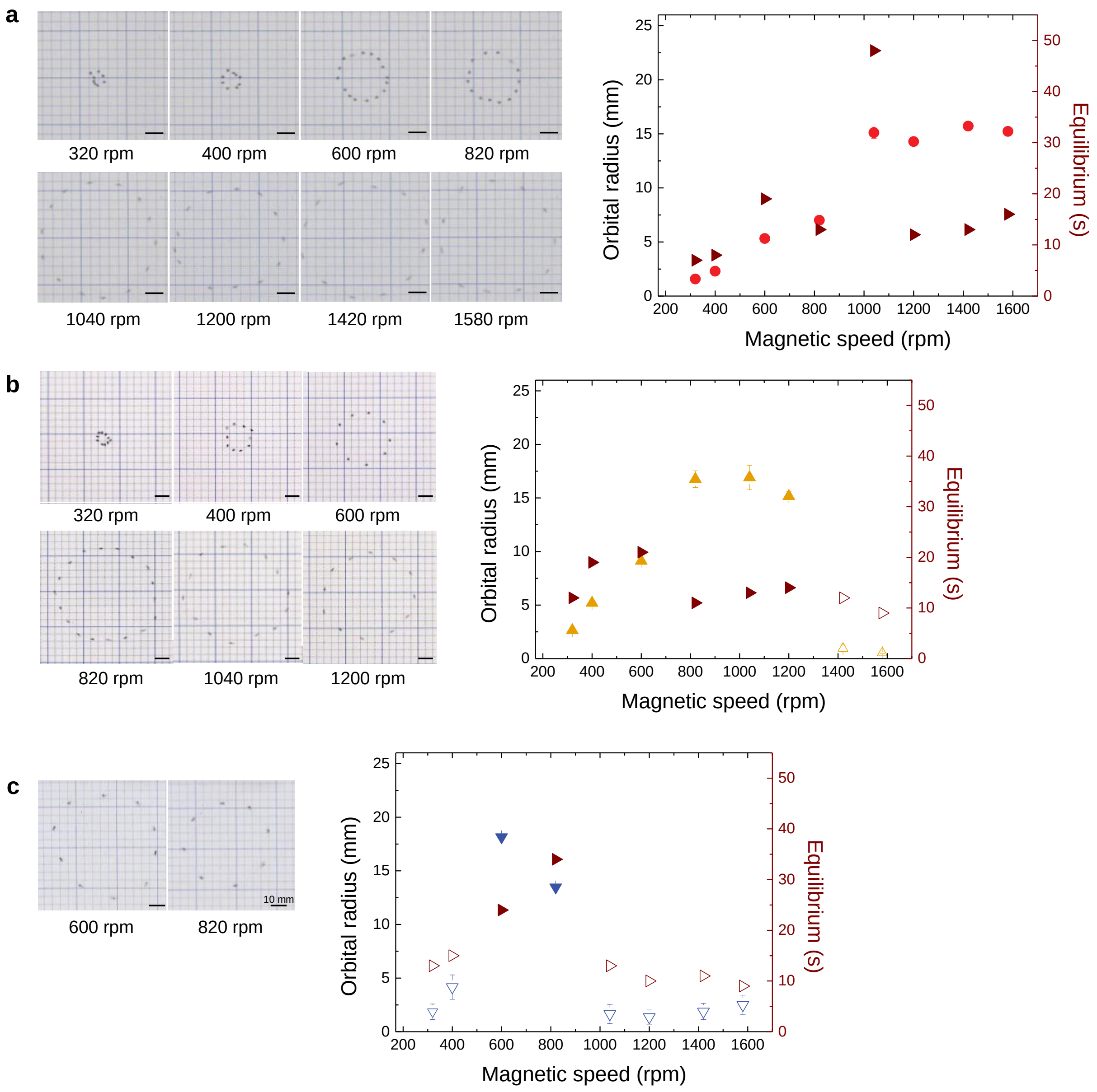

Supplementary Figure 8. Effects of aspect ratio of the spinbots. (a)-(c) The orbital trajectory image (left) and orbital radius diagram (left). The open symbols represent capriciously revolving. (a) AR-2, (b) AR-3, and (c) AR-4. Error bars represent the standard deviation (n = 3).

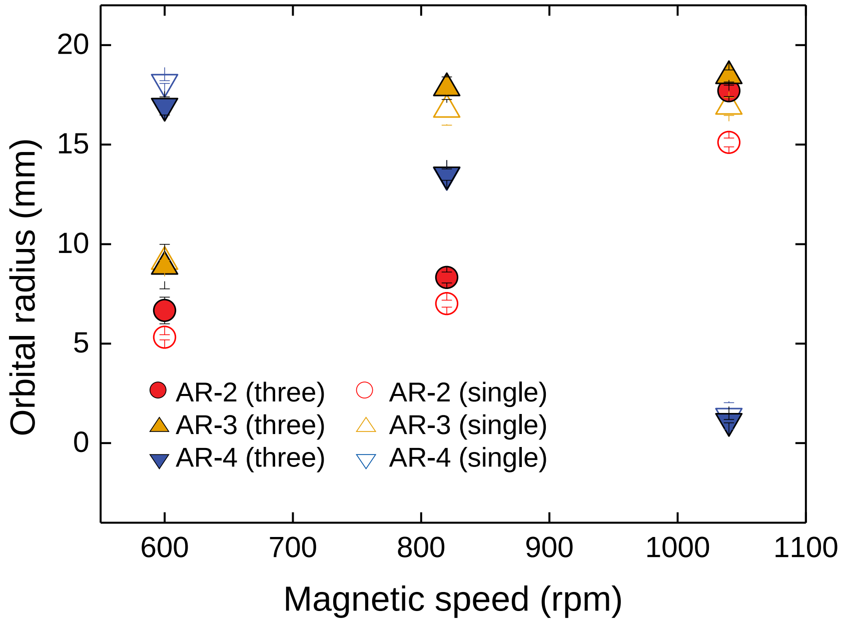

Supplementary Figure 9. Multi-object orbital maneuver. Comparison between three bodies and a single body manipulation. Note that interactions between the spinbots are not dominant in a multi-body system, confirmed by actual observational radius. Error bars represent the standard deviation (n = 3).

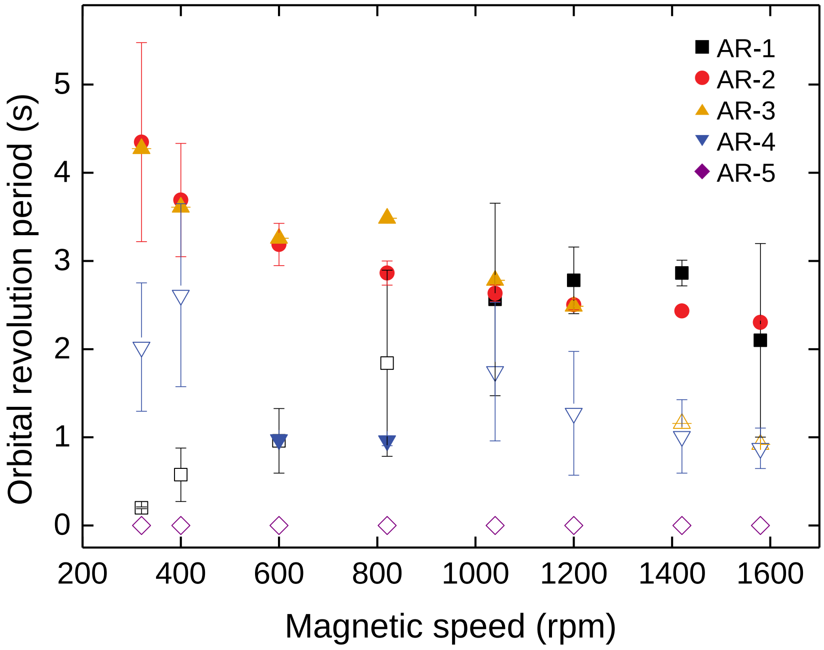

Supplementary Figure 10. Orbital period. The period of orbital revolution is calculated for the orbital velocity according to rotational speed. The spinbots of open symbols actuate non-uniformly. Error bars represent the standard deviation (n = 3).

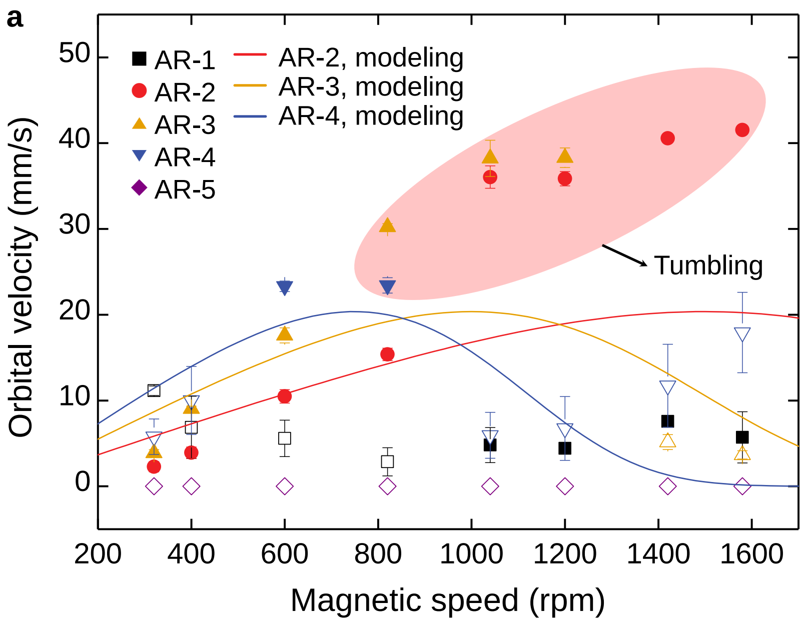

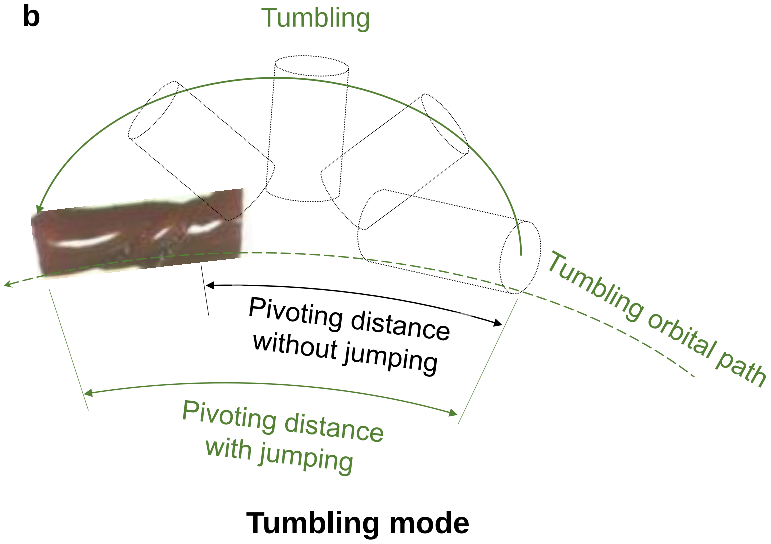

Supplementary Fig. 11. Accelerated orbital velocity when the spinbot is in tumbling mode which is pivoting behavior with jumping. (a) The orbital velocity according to different aspect ratios of the spinbot and varying rotational speeds of magnets. Schematics showing (b) the tumbling mode along an orbital path. Error bars represent the standard deviation (n = 3).

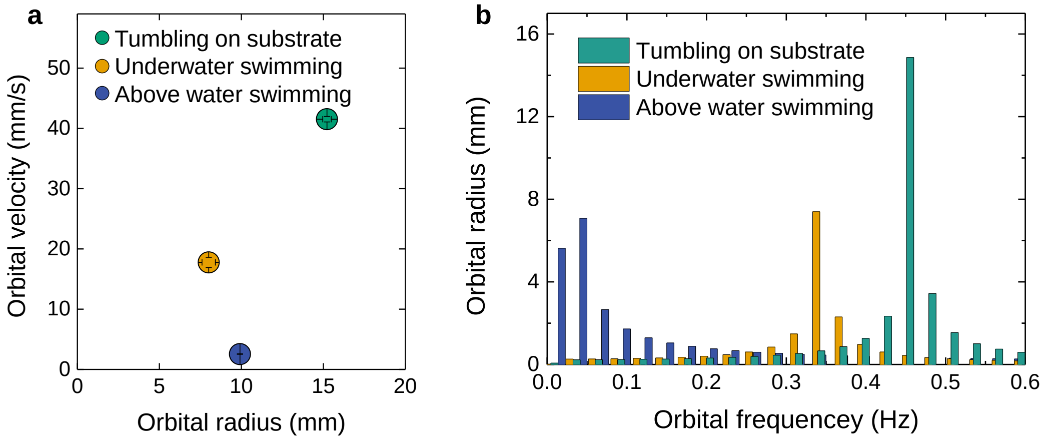

Supplementary Figure 12. Amphibious functionality. 3D dense structure of the spinbot is reasonable to underwater swimming movements. The AR-2 spinbot has three orbiting actuations at 1580 rpm. (a) The velocity of an underwater swimming spinbot at 2 mm of depth is 7 times faster than a spinbot swimming above water. Error bars represent the standard deviation (n=3). (b) Orbital trajectory of sinusoid in Fig. 4d is transferred to orbital frequency and orbital radius by Fast Fourier transform.

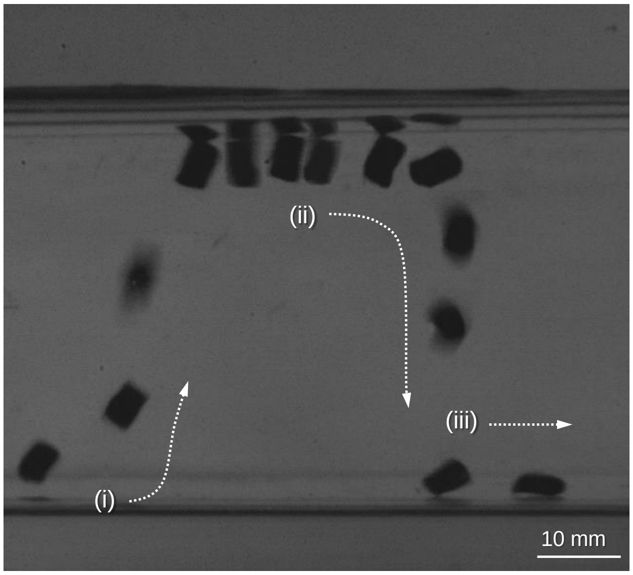

Supplementary Figure 13. Reversible actuation of underwater swimming and swimming beneath the surface of water. The spinbot continues to swim by hierarchical magnetomotility with 600 rpm alongside the additional static magnetic force emanating from the surface of water. Swimming up to vertical direction (i) by neodymium magnet, reversibly diving back to underwater by removal of the magnetic force (ii), and continuing underwater swimming (iii).

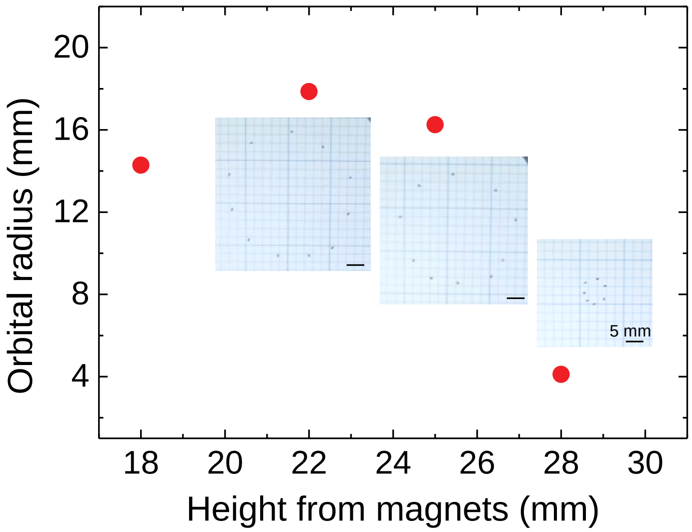

Supplementary Figure 14. Orbital radius by varying vertical distance between soft robot and magnet. The orbital radius of AR-2 has no significant changes at 1000 rpm by increasing the distance between soft robot and magnets up to 25 mm. When the height is larger than 28 mm, AR-2 finally stops rotation in the course of the revolution.

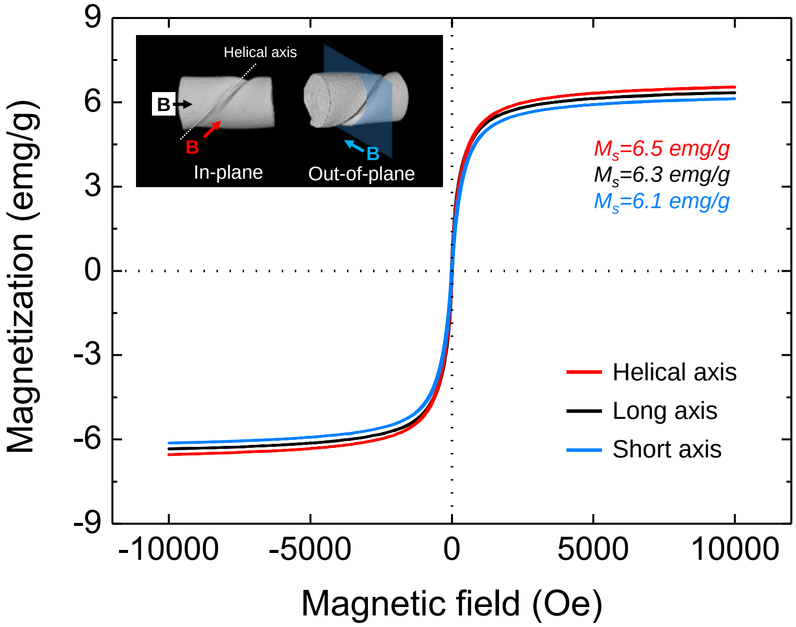

Supplementary Figure 15. Magnetization curves of twisted structure of the TPU-Fe_3O_4 composite at 300 K. The magnetization is not significantly changed depending on the direction of the external applied magnetic field. Magnetic property of the helical axis is measured under in-plane parallel magnetic field. Note that the measurement deviation of the saturated magnetization (M_s) is caused by a positional error during adjusting the center in VSM (Quantum Design, MPMS3 SQUID VSM). Helical geometry effect is more dominant on orbital revolution than different anisotropy of the magnetic property as the difference in M_s distribution is not significant.

Supplementary Note 1. The dispersion state of nanoparticles

Iron oxide nanoparticles that z-averaged diameter d_z is determined by dynamic light scattering (DLS) fitting by lognormal distribution[1,2], which can be expressed as

$$p(x) = \frac{1}{x\sigma\sqrt{2\pi}} \exp\left[-\frac{(\ln x-\mu)^2}{2\sigma^2}\right] \ (x > 0) \tag{1}$$

where x is the diameter of nanoparticle, μ is the mean value, and σ is the standard deviation. The DLS measurement provides hydrodynamic diameter (d_h= 13.7 nm) and polydispersity index (p= 0.06) where p is $(\sigma/\mu)^2$. The z-averaged diameter d_z is calculated to be 16.2 nm by using the number-averaged diameter $< d >$ and the value of z.

$$d_z = < d > \frac{[z+3][z+2]}{[z+1]^2} \tag{2}$$

$$z = \frac{1}{p} - 1 \tag{3}$$

A dispersion state of 10 wt% magnetic nanoparticles in thermoplastic polyurethane (TPU) matrices is measured by fractal analysis[3,4]. The fractal geometry quantifies complexity of objects and local connected fractal dimension α examines connected cluster in a scaling box of size ε. One-dimension describes α= 1 with a straight line, whereas α= 2 is two-dimensional of a completely filled area. The relationship expressed as

$$M(\varepsilon) \propto F_\varepsilon^\alpha \tag{4}$$

$$\alpha = \frac{\log[M(\varepsilon)]}{\log(\varepsilon)} \tag{5}$$

where F is the mass pre-factor and α is the exponent. Lacunarity λ with correlations of discrepancy of the expected fractal dimension follows as

$$\lambda = \overline{\left(\frac{\alpha}{\bar{\alpha}} - 1\right)^2} \tag{6}$$

The local mass scaling property is calculated through conversion from the TEM micrograph in Fig. 1b into the local connected fractal dimension using NIH ImageJ software. The 10 wt% polymer nanocomposite film reveals α= 1.76 with λ= 0.09. The results of calculated local complexity indicate homogeneous dispersion of iron oxide nanoparticles in TPU matrices.

Supplementary Note 2. Magnetic properties of the composite

In hysteresis loops of magnetic nanoparticles, magnetite (Fe_3O_4) appears to have no significant remanence (Supplementary Fig. 1c). Saturated magnetization, M_s is measured to be 60 emu g^{-1} and 9 emu g^{-1} for Fe_3O_4 and the composite film (TPU-Fe_3O_4), respectively. Coercivity, H_c of 28 Oe is measured for both the magnetite and the composite film. We confirm that the magnetite and nanocomposites appear with superparamagnetism effectively in consideration of their coercivity, particle size, and crystallite size (Supplementary Fig. 1d). Crystallite size of magnetite is calculated with 8.6 nm and 8.7 nm for 10 wt% composite by Scherrer equation[5]. When the crystallite size is compared with d_z of hydrodynamic volume and the visualized image of TEM, the composite obtains single domain with superparamagnetism[6,7]. Polydispersity of the iron oxide nanoparticles (p= 0.06) affects the coercivity value due to relatively week ferromagnetism of larger than 10~20 nm of particle diameter.

Supplementary Note 3. Preparation of the spinbots

TPUs with their high strain-to-failure (650 %) are suitable for construction of their three-dimensional structures by twisting of two-dimensional films. Hence, the stretchable composite film is physically twisted and fixed by heat treatment to prepare for the assembly of magnetically responsive helical 3D soft robots. As heavy magnetic microparticles are susceptible to precipitation through gravitational force due to their high density, we select 10 nm Fe_3O_4 nanoparticles for their dispersion quality in a solvent. The sedimentation rate of nanoscale particles is noticeably slow in a good solvent due to the force balance between a sedimentation force induced by gravitational force and a drag force present by cause of the buoyancy force[8]. In addition, small nanoparticles can be dispersed in polymer matrices via the rapid precipitation technique[9].

The optimum helical diameter of the spinbots is determined after testing various twisting and supertwisting conditions so that the 3D spinbots can be constructed without void formation. When a void is present in the spinbots, the spinbots are not able to follow the high speed spin of the magnetic field, leading to absence of rotation or the immediate stop of rotational movement (Supplementary Fig. 2c). Supertwisting occurs when the twisting number is greater than 20. Without the supertwisting process, constructed spinbots are asymmetric and hollowed as evident from the OM micrographs. After the supertwisting, the spinbots are stretched to secure uniform diameter. When the stretched length is too long, it is difficult to observe the effect of the aspect ratio due to the too large helical pitch. Optimum conditions require 30 times of twisting and 3 cm stretching to generate dense structures with 0.3 mm of diameter which allow the spinbots to rotate and revolve hierarchically

Supplementary Note 4. Orbit analysis of the spinbots

The clockwise rotating magnetic field triggers clockwise rotating and results in a counterclockwise revolving motion for twisted soft robots. Here, the counterclockwise revolution is generated by the centrifugal force from a slight mismatch of the center of gravity by the non-cylindrical structure of helical geometry. Regardless of the handedness, clockwise rotating

generates outward centrifugal force and the spinbots revolve in a counterclockwise direction through the same mechanism. The orbital radius of the right handed AR-2 is comparable with the left handed AR-2 (Supplementary Fig. 4b).

We confirm that the magnetic field vector determines the lift angle of the spinbots, generating trimodal rotations. The direction of magnetic flux density is changed to perpendicular from horizontal as the spinbot moves from the middle of the two magnets (center of rotating axis) to the center of the magnet (Supplementary Fig. 5b). Orbital radii are regulated by the rotation modes, originating from the lift angles. To confirm this effect, we physically relocate the rotating spinbots by external force (Supplementary Fig. 5c). At 1420 rpm, the AR-3 spinbot revolves erratically due to slippage. In this system, a centrifugal force generated by the spinbot rotation alone is insufficient. Hence, the centrifugal force is replaced with an external force to physically relocate the spinbot toward an outward direction. This relocation changes the lift angle and consequently the spinbot changes its rotational mode from rotating to tumbling thereby achieving a large orbital radius and faster orbital velocity.

Supplementary References

1. Zhang, J. & Buffle, J. Kinetics of hematite aggregation by polyacrylic acid: importance of charge neutralization. *J. Colloid Interface Sci.* **174,** 500–509 (1995).
2. Kim, S.-K., Nguyen, N. A., Wie, J. J. & Park, H. S. Manipulating the glass transition behavior of sulfonated polystyrene by functionalized nanoparticle inclusion. *Nanoscale* **7,** 8864–8872 (2015).
3. Landini, G. & Rippin, J. W. How important is tumour shape? quantification of the epithelial–connective tissue interface in oral lesions using local connected fractal dimension analysis. *J. Pathol.* **179,** 210–217 (1996).
4. Landini, G., Murray, P. I. & Misson, G. P. Local connected fractal dimensions and lacunarity analyses of 60° fluorescein angiograms. *Invest. Ophthalmol. Vis. Sci.* **36,** 2749–2755 (1995).
5. Li, L., Yang, Y., Ding, J. & Xue, J. Synthesis of magnetite nanooctahedra and their magnetic field-induced two-/three-dimensional superstructure. *Chem. Mater.* **22**, 3183–3191 (2010).
6. Kim, D. *et al.* Synthesis of uniform ferrimagnetic magnetite nanocubes. *J. Am. Chem. Soc.* **131**, 454–455 (2009).
7. Li, Q. *et al.* Correlation between particle size/domain structure and magnetic properties of highly crystalline Fe3O4 nanoparticles. *Sci. Rep.* **7**, 9894 (2017).
8. Park, J. E. et al. Magnetomotility of untethered helical soft robots. *RSC Adv.* **9,** 11272–11280 (2019).
9. Mackay, M. E. et al. General strategies for nanoparticle dispersion. *Science* **311,** 1740–1743 (2006).
