# Supplementary material for: On-demand orbital maneuver of multiple soft robots via hierarchical magnetomotility
Source: Nat Commun. 2019 Oct 18;10:4751. doi: 10.1038/s41467-019-12679-4 (PMC6802085; doi:10.1038/s41467-019-12679-4)
Supplement: Supplementary file 2 — Description of Additional Supplementary Files [file 41467_2019_12679_MOESM2_ESM.pdf]

# Description of Additional Supplementary Files

**Supplementary Movie 1.** Three rotational modes via rotational magnetomotility

**Supplementary Movie 2.** Reversible orbital maneuver of spinbot

**Supplementary Movie 3.** Regulation of orbital radius

**Supplementary Movie 4.** Regulation of orbital velocity

**Supplementary Movie 5.** Orbital maneuverability in the three-body system

**Supplementary Movie 6.** Conformal navigation of spinbots

**Supplementary Movie 7.** Multifunctionality in spinbots

**Supplementary Movie 8.** Multiple soft robots for collective behavior

**Supplementary Movie 9.** Directional control of swimming
